# Advanced Glycation End Products and Genetic Polymorphism in the Pathogenesis of Microvascular Complications in Type 2 Diabetes Mellitus: A Narrative Review

**DOI:** 10.7759/cureus.109935

**Published:** 2026-05-30

**Authors:** Rajlaxmi Sarangi, Sweta Singh, Jyoti Prakash Sahoo, Amrita Satpathy

**Affiliations:** 1 Department of Biochemistry, Kalinga Institute of Medical Sciences, Bhubaneswar, IND; 2 Department of Pharmacology, Kalinga Institute of Medical Sciences, Bhubaneswar, IND

**Keywords:** advanced glycation end-products, ager gene, diabetic microvascular complications, endothelial dysfunction, extracellular matrix protein, nuclear factor kappa-β, receptor for advanced glycation end products, redox signaling, single-nucleotide polymorphism (snp), type 2 diabetes mellitus

## Abstract

Patients with type 2 diabetes mellitus often experience microvascular complications, including diabetic retinopathy, nephropathy, and neuropathy. Despite advances in glucose-lowering treatments, these complications remain a significant clinical and financial burden worldwide. Their pathogenesis is influenced by genetic polymorphism and advanced glycation end products (AGEs). Chronic metabolic stress leads to the formation of AGEs via oxidative and nonenzymatic glycation. AGEs bind to the AGE receptor (RAGE) and actively cause microvascular damage. This occurs through endothelial dysfunction, amplification of oxidative stress, protein cross-linking, and inflammatory activation. Genes controlling AGE production, AGE-RAGE signaling, oxidative stress responses, endothelial nitric oxide bioavailability, and extracellular matrix remodeling affect individual susceptibility to AGE-mediated microvascular damage. Elevated AGE load and genetic polymorphisms influence metabolic memory, microvascular injury, and redox signaling. This review article outlines the molecular mechanisms involving AGEs, genetic polymorphisms, and microvascular complications. Understanding AGE biology and genetic risk analysis could support management of diabetic microvascular complications and foster personalized medicine and preventive approaches beyond traditional glucose-centric frameworks.

## Introduction and background

Type 2 diabetes mellitus (T2DM) is a chronic metabolic disease triggered by an absolute or relative deficit in insulin secretion, activity, or both [1,2]. Hyperglycemia is a hallmark of T2DM. Chronic uncontrolled hyperglycemia often leads to microvascular complications like diabetic neuropathy, nephropathy, and retinopathy [1-3]. These complications persist in the current world, despite advancements in treatment approaches and good medication adherence [2,4]. It implies that more intricate molecular processes than just persistent hyperglycemia are involved [3,4].

Advanced glycation end products (AGEs) have crucial roles in the development of diabetic microangiopathy. Prolonged hyperglycemia and increased oxidative stress facilitate the formation of AGEs. Reducing sugars and other saccharide derivatives react nonenzymatically with lipids, peptides, amino acids, and nucleic acids to produce AGEs [5,6]. AGEs compromise the normal capillary function by increasing vascular stiffness, altering the extracellular matrix (ECM), and thickening basement membranes [5,7-9]. AGEs bind the receptor for AGE (RAGE) and initiate intracellular signaling pathways that mediate oxidative stress and endothelial dysfunction [7,9,10]. The AGE-RAGE signaling axis activates nuclear factor-κB (NF-κB)-mediated transcription of proinflammatory cytokines, adhesion molecules, and growth factors. It leads to microvascular injury and inflammation in target organs, i.e., the kidney, retina, and peripheral nerves [11-14]. Recent studies have found positive associations between elevated circulating and tissue AGE levels and the severity of microvascular complications [11,13-16].

The clinical course of T2DM shows interindividual variation. Around one-fifth of the diabetics develop microvascular complications regardless of glycemic exposure and pharmacotherapy [15,17,18]. This observation has prompted us to consider the role of genetic susceptibility in modulating disease risk. Genetic polymorphisms affecting genes involved in AGE-RAGE signaling have been recognized as critical determinants of microvascular damage [8,14,19]. Polymorphic variants in RAGE, endothelial nitric oxide synthase (eNOS), and aldose reductase enzyme genes are associated with impaired vascular homeostasis, altered inflammatory responses, and increased oxidative stress in diabetic individuals [20,21].

The interactions between AGE-RAGE signaling and genetic polymorphisms provide a mechanistic framework for the heterogeneity of microvascular complications. It highlights the limitations of therapeutic approaches that focus solely on glycemic status [22-24]. In this context, elucidating the roles of AGEs and genetic polymorphisms in the pathogenesis of microvascular complications is the need of the hour for developing precision-based preventive and therapeutic strategies. This review plans to describe the molecular mechanisms linking AGEs and genetic polymorphisms to microvascular complications in T2DM, with an emphasis on their clinical implications and future research directions.

## Review

T2DM and the spectrum of microvascular complications

Chronic hyperglycemia in T2DM results from impaired insulin secretion, increased insulin resistance, and excess hepatic glucose production [2,25]. Genetic predisposition and other factors such as physical inactivity, chronic stress, and obesity hasten the progression of disease [25,26]. By 2024, 589 million adults had T2DM, and by 2050, there will be 853 million cases of T2DM worldwide. This 45% increase in cases can be attributed to population growth, aging, and urbanization [27]. The burden of T2DM is further enhanced by its complications. The microvascular complications reflect the progression and severity of the disease [18,28]. These complications include diabetic retinopathy, nephropathy, and neuropathy [3]. Microvascular complications are triggered by structural and functional alterations in small blood vessels, such as capillaries, arterioles, and venules. These issues lead to tissue ischemia, cellular dysfunction, and organ failure [18,29].

The most prevalent microvascular complication related to T2DM is diabetic retinopathy [6,30]. Its hallmark is progressive microvascular damage in the retinal circulation. This damage includes thickening of the capillary basement membrane, loss of pericytes, formation of microaneurysms, and pathological neovascularization, which eventually leads to retinal ischemia and visual impairment. Chronic hyperglycemia, inflammation, oxidative stress, and altered angiogenic signaling in the retinal microenvironment all contribute to the pathophysiology of diabetic retinopathy [23,31,32].

Diabetic nephropathy is another microvascular complication of T2DM [3]. Diabetic individuals with albuminuria and/or a decrease in estimated glomerular filtration rate are said to have diabetic nephropathy [33,34]. The American Diabetes Association states that an albumin-to-creatinine ratio above 30 mg/g is regarded as pathological albuminuria [33]. Many processes, including oxidative stress, fibrosis, hyperinflammation, and glomerular hemodynamics (i.e., glomerular hyperfiltration), contribute to the etiology of diabetic kidney disease [33,34]. In T2DM, albuminuria and co-occurring retinopathy are strong indicators of microvascular damage and, therefore, strongly predictive of diabetic nephropathy [33-35].

Diabetic neuropathy includes peripheral and autonomic nerve conditions that greatly reduce quality of life and raise the risk of limb amputation and foot ulcers [6,36,37]. Nerve ischemia, axonal degeneration, and demyelination seen in diabetic neuropathy are largely caused by microvascular inadequacy of the vasa nervorum, metabolic disorders, and oxidative stress [36-38].

There is significant interindividual variation concerning the emergence and progression of microvascular complications of T2DM, with some diabetics remaining remarkably protected from these complications despite their chronic diabetes. Others, on the other hand, experience serious complications at an early stage of the illness [15,39]. This heterogeneity underscores the role of nonglycemic factors in regulating microvascular risk, including genetic vulnerability, epigenetic changes, and differential activation of pathogenic metabolic pathways [18,26]. As a result, long-term hyperglycemia, together with intrinsic biological factors that control vascular integrity and repair mechanisms, is now regarded as the combined cause of microvascular complications [15,24,26,30].

Pathophysiology of microvascular complications

Chronic hyperglycemia causes metabolic, hemodynamic, and inflammatory changes that cause the microvascular complications of T2DM [18,25]. Persistent hyperglycemia causes tissue ischemia, cellular dysfunction, and organ-specific damage by compromising microvascular structure and function [28,29]. Increased generation of reactive oxygen species (ROS) results from increased intracellular glucose flux [40]. The polyol pathway, hexosamine biosynthesis, protein kinase C (PKC) activation, and AGE production are linked to microvascular injury induced by oxidative stress [5,40].

Activation of the polyol pathway in hyperglycemic conditions increases the conversion of glucose to sorbitol via aldose reductase, using nicotinamide adenine dinucleotide phosphate (NADPH), lowering the antioxidant capacity of cells [41]. Sorbitol and fructose accumulation in microvascular tissues, especially in the kidney, retina, and peripheral nerves, causes osmotic stress and endothelial dysfunction [41,42]. Hyperglycemia-induced diacylglycerol accumulation plays a crucial role in microvascular dysfunction by altering capillary permeability, blood flow control, and ECM production, and PKC activation [43]. PKC-mediated signaling increases production of vascular endothelial growth factor (VEGF), endothelin-1, and TGF-β. These factors contribute to aberrant angiogenesis, vasoconstriction, and fibrosis, which collectively facilitate microvascular complications of T2DM [41,43,44].

Another important factor underlying diabetic microangiopathy is the generation and accumulation of AGEs. AGEs alter the structural proteins in the ECM and basement membrane, increasing capillary wall thickness and vascular stiffness [5-8,10]. AGE-RAGE interaction activates intracellular inflammatory cascades, such as NF-κB signaling, thereby prolonging oxidative stress, endothelial damage, and chronic inflammation in microvascular beds [12-16]. Increased vascular permeability, enhanced leukocyte adhesion, and decreased nitric oxide (NO) bioavailability are all indicators of endothelial dysfunction [14,20].

Endothelial cell damage impairs microcirculation's normal autoregulatory mechanisms, making tissues more prone to hypoxia and ischemia injury [21,45]. Furthermore, organ-specific signs of microvascular damage are exemplified by ischemia of the vasa nervorum, podocyte damage in glomeruli, and pericyte loss in retinal capillaries [45,46]. Through epigenetic processes, including DNA methylation and histone modifications, hyperglycemia induces long-lasting changes in gene expression. This phenomenon is known as "metabolic memory." It causes vascular damage even after glycemic correction [22,23]. The pathophysiology of microvascular complications in T2DM results from chronic hyperglycemia, inflammation, oxidative stress, endothelial dysfunction, and maladaptive cellular signaling pathways [20-22]. Understanding these pathways is crucial for formulating targeted therapeutic approaches to halt the progression of diabetic microangiopathy beyond traditional glycemic management [14,16,23,24,30].

Formation and types of AGEs

AGEs can originate from both endogenous and external sources [6,7,9]. Prolonged hyperglycemia and increased oxidative stress facilitate the nonenzymatic reaction to generate AGEs from free amino groups of proteins, lipids, nucleic acids, and reducing sugars [6,46,47]. AGEs are formed in three stages through the Maillard reaction [7,9,46,47]. The first stage generates unstable Schiff bases (e.g., aldimines) from the free carbonyl groups of reducing sugars and the free amino acids (e.g., lysine, arginine, and cysteine) of proteins [6,47]. The second stage converts the unstable aldimine to a more stable Amadori product, such as glycated hemoglobin (HbA_1c_), via an Amadori rearrangement [7,46,47]. In the third stage, this Amadori product (i.e., HbA_1c_) changes its tertiary and quaternary structure through oxidation and reduction to create irreversible AGEs [7,9,46]. AGEs are especially pertinent to the pathophysiology of T2DM and its microvascular complications [6,7,9]. Chronic hyperglycemia, oxidative stress, and carbonyl stress accelerate the formation of AGEs [7,46,47].

Cigarette smoking, excess consumption of carbohydrates and hypercaloric diet, cooking at high temperatures, and leading a sedentary lifestyle encourage the development of AGEs [46,48]. Food-derived or dietary AGEs (dAGEs) contribute significantly to the systemic AGE burden [48,49]. Changes in cell signaling transduction, insulin-mediated metabolic responses, reduced innate defenses, insulin resistance, oxidative stress, and increased serum AGE levels have all been linked to increased dAGE consumption [46-49]. Individuals with renal impairment exhibit greater intestinal absorption of dAGE [49,50].

AGEs include a broad range of structurally diverse compounds, including cross-linking and non-cross-linking moieties, as well as fluorescent and nonfluorescent compounds [51,52]. Pentosidine, glucosepane, carboxymethyl lysine, and carboxyethyl lysine are some examples of AGEs [53]. In the ECM, these AGEs accumulate within collagen, laminin, and elastin, altering tissue architecture, disrupting normal cellular connections, and stiffening ECM proteins [51-53]. AGE formation is linked to the onset and progression of microvascular complications because the persistent accumulation of AGEs in tissues and the circulation serves as a metabolic substrate for downstream AGE-RAGE signaling [7-9,46,47,53].

Molecular mechanisms of AGE-mediated vascular injury

AGE accumulation in the ECM proteins results in excessive protein cross-linking and altered ECM architecture [51,54]. These events compromise capillary integrity and permeability by increasing vascular stiffness, decreasing arterial compliance, and impairing normal interactions between endothelial cells and the basement membrane [52-54]. Additionally, AGE-modified matrix proteins are resistant to proteolytic degradation, leading to a gradual thickening of the basement membrane, a hallmark of diabetic microangiopathy [8,54]. RAGE is widely expressed on macrophages, pericytes, vascular smooth muscle cells, and endothelial cells [14,24].

AGE-RAGE interactions trigger the activation of NF-κB, Janus kinase/signal transducer and activator of transcription (JAK/STAT), and mitogen-activated protein kinases (MAPKs) [13,14]. These processes lead to an increased transcription of proinflammatory and proatherogenic genes [13-15,20]. NF-κB activation is a key mechanism via which AGE-RAGE signaling perpetuates vascular damage [13,14]. Leukocyte recruitment and endothelial inflammation are facilitated by NF-κB-dependent transcription, which increases the expression of tumor necrosis factor-α (TNF-α), interleukin-6 (IL-6), monocyte chemoattractant protein-1 (MCP-1), vascular cell adhesion molecule-1 (VCAM-1), and intercellular adhesion molecule-1 (ICAM-1) [55,56]. RAGE activation creates a positive feedback loop by increasing its own expression and cellular sensitivity to AGEs, and maintaining inflammatory signaling even in euglycemic individuals [19,57].

AGE-induced vascular damage is also strongly influenced by oxidative stress. Through activation of NADPH oxidase and mitochondrial dysfunction, the AGE-RAGE interaction promotes excessive ROS production, resulting in redox imbalance within vascular cells [56,58]. In addition to directly harming cellular macromolecules, elevated ROS levels also increase the production of AGEs and the expression of RAGE, perpetuating a vicious cycle of inflammatory and oxidative damage [10,58].

AGE formation significantly worsens endothelial dysfunction, a hallmark of diabetic vascular disease. AGEs lower NO bioavailability by decreasing eNOS activity and encouraging superoxide-mediated NO scavenging [20,21]. Concurrently, AGEs promote the synthesis of prothrombotic and vasoconstrictive mediators such as endothelin-1 and plasminogen activator inhibitor-1 [13,14]. AGE-RAGE signaling pathways cause aberrant ECM proliferation in vascular smooth muscle cells [55,57]. Additionally, renal and retinal microvascular fibrosis is accelerated by AGE-mediated activation of TGF-β signaling, thereby aggravating diabetic nephropathy and retinopathy, respectively [59].

AGE-RAGE signaling pathway and inflammation

RAGE is a multiligand pattern-recognition receptor of the immunoglobulin superfamily regulated by the AGER gene. It is constitutively expressed at low levels in most tissues but is markedly upregulated under conditions such as chronic inflammation, oxidative stress, and hyperglycemia [60]. When AGEs bind to RAGE, an intricate network of intracellular signaling cascades is initiated, intensifying oxidative and inflammatory responses in vascular cells. Proinflammatory genes undergo transcriptional reprogramming as a result of this interaction, which activates several kinase pathways, including MAPK, extracellular signal-regulated kinase (ERK1/2), and c-Jun N-terminal kinase [61,62]. AGE-RAGE signaling causes sustained activation of NF-κB, a master regulator of inflammatory gene expression. Inhibitor of NF-κB alpha is phosphorylated and degraded by AGE-induced RAGE activation, which facilitates NF-κB nuclear translocation and subsequent transcription of cytokines, chemokines, adhesion molecules, and growth factors [60,62]. Additionally, RAGE expression is upregulated by NF-κB activation, creating a positive feedback loop that sustains inflammatory signaling even in the absence of continuous glycemic insult. This process has a role in metabolic memory [62]. Through activation of NADPH oxidase and dysfunction of the mitochondrial electron transport chain, the AGE-RAGE interaction leads to excessive ROS generation. In addition to directly damaging cells, this oxidative burst also acts as a second messenger, increasing NF-κB activity and activating inflammatory genes [60-62]. This aggravates endothelial and microvascular damage by promoting DNA damage, lipid peroxidation, and post-translational protein modification [60,61].

TNF-α, IL-6, interleukin-1β, and MCP-1 are inflammatory mediators that are triggered by AGE-RAGE signaling. These mediators aid in leukocyte recruitment, macrophage activation, and vascular inflammation. Additionally, increased expression of endothelial adhesion molecules, such as VCAM-1, ICAM-1, and E-selectin, promotes transendothelial migration of circulating immune cells into the vascular wall [55,61]. In diabetic microvascular beds, AGE-RAGE-mediated inflammation activates profibrotic signaling pathways, particularly through the overexpression of connective tissue growth factor and TGF-β, leading to ECM buildup and tissue fibrosis [60-62]. This inflammatory-fibrotic axis accelerates capillary obliteration, basement membrane thickening, and mesangial expansion in renal and retinal tissues, which hastens the development of diabetic nephropathy and retinopathy [56,60,62].

AGEs in diabetic retinopathy, nephropathy, and neuropathy

AGEs are powerful mediators of oxidative stress, cellular dysfunction, and vascular inflammation. Hence, they contribute to the development and progression of diabetic microvascular complications [46,63]. AGE accumulation in retinal capillary basement membranes, endothelial cells, and pericytes causes severe microvascular dysfunction in diabetic retinopathy. Retinal microcirculation is jeopardized when AGEs cross-link with ECM proteins, thickening the basement membrane and reducing capillary flexibility [7,47]. Proinflammatory and proangiogenic signaling pathways, particularly NF-κB and MAPKs, are activated by AGEs via RAGE in retinal endothelial cells, leading to elevated VEGF production and pathological neovascularization [5,64].

In individuals with diabetic nephropathy, AGEs accumulate in the glomerular basement membrane (GBM), mesangial matrix, and podocytes [63,64]. Increased GBM thickness and mesangial expansion are caused by AGE-induced cross-linking of collagen and laminin [46,47]. AGE-RAGE interaction triggers inflammatory and profibrotic pathways, such as NF-κB and TGF-β signaling, which promote ECM deposition on podocytes and glomerulosclerosis [5,63]. AGEs damage podocytes, compromise cytoskeletal integrity, and promote apoptosis, thereby disrupting the glomerular filtration barrier and increasing albuminuria [46,63-65]. AGE-mediated oxidative stress and inflammation worsen tubulointerstitial fibrosis and renal dysfunction, which lead to the development of end-stage renal disease [47,64].

The severity of diabetic neuropathy is directly correlated with AGE accumulation in peripheral nerves [63,65]. AGEs cause chronic neuronal ischemia and hypoxia by decreasing NO bioavailability and inducing endothelial dysfunction, thereby impairing neural blood flow [46,63,64]. AGEs alter neuronal cytoskeletal proteins and myelin components. It leads to impaired axonal transport and nerve conduction velocity [5,63-65]. Axonal degeneration and demyelination are caused by oxidative stress, mitochondrial failure, and inflammatory cytokine release when AGE-RAGE signaling is activated in Schwann cells and neurons [46,47,64].

Clinical implications of genetic polymorphism in T2DM

T2DM is a polygenic disease in which numerous single-nucleotide polymorphisms (SNPs) collaborate to trigger altered glucose metabolism, insulin resistance, decreased insulin production, and β-cell dysfunction [66,67]. Over the past two decades, hundreds of loci linked to diabetes have been identified through genome-wide association studies (GWAS), implicating genes in inflammatory pathways, incretin signaling, lipid metabolism, pancreatic β-cell formation, and mitochondrial function [66]. In addition to increasing the risk of disease, these polymorphisms also affect the onset of metabolic disorders and response to treatment [67].

Genetic polymorphisms are important in predicting susceptibility to microvascular and macrovascular complications of T2DM [66]. Variants in the genes encoding superoxide dismutase (SOD), eNOS, aldose reductase, and components of the renin-angiotensin-aldosterone system modify tissue vulnerability to oxidative and hemodynamic stress induced by hyperglycemia, thereby regulating microvascular damage [66,68]. Similarly, increased inflammatory responses and accelerated vascular damage in diabetes have been linked to polymorphisms in genes involved in AGE formation, detoxification, and RAGE-mediated signaling [66-68]. Clinical risk stratification using genetic polymorphism data can identify high-risk individuals early and provide targeted care [66,67].

Polymorphisms of genes related to AGE formation and metabolism

Genes regulating glucose metabolism, carbonyl stress, oxidative balance, and cellular defense systems also control the production, accumulation, and detoxification of AGEs [47,69]. AGE production is primarily influenced by genes related to glucose and carbonyl metabolism [7,70]. Under hyperglycemic conditions, aldose reductase (AKR1B1), a crucial enzyme of the polyol pathway, catalyzes the reduction of glucose to sorbitol, thereby indirectly increasing intracellular concentrations of reactive dicarbonyl compounds, such as methylglyoxal, which are powerful precursors of AGE formation [7,47,69,70]. The pathogenic significance of the AKR1B1 gene in AGE biology has been highlighted by its polymorphisms, which have been linked to increased enzymatic activity, increased carbonyl stress, and an increased risk of diabetic microvascular complications [69,70].

The glyoxalase system, which consists of glyoxalase 1 (GLO1) and glyoxalase 2, is primarily responsible for the detoxification of reactive dicarbonyl molecules [9,71]. GLO1 prevents excessive AGE production by catalyzing the transformation of methylglyoxal into S-D-lactoylglutathione [9,47,71]. Methylglyoxal accumulation and rapid glycation of proteins, lipids, and nucleic acids are brought about by decreased expression and functional polymorphisms in the GLO1 gene [71,72]. It is frequently observed in T2DM [9,47]. Reduced GLO1 activity has been linked to higher AGE load and microvascular damage in diabetes, according to experimental and clinical research [9,47,69-72]. Variants in the genes of antioxidant enzymes, such as catalase, glutathione peroxidase (GPX1), and SOD types 1 and 2 (SOD1 and SOD2), disrupt cellular redox homeostasis and promote oxidative glycoxidation processes that favor the development of AGEs [9,71,72].

Systemic AGE load is influenced by genes involved in AGE breakdown and clearance, as well as production and detoxification pathways [69,71]. AGE uptake and elimination are facilitated by scavenger receptors and AGE-binding proteins that are present in renal tissues, endothelial cells, and macrophages [71,72]. Systemic accumulation of circulating AGEs and tissue deposition result from impairment of renal elimination mechanisms, whether genetically determined or associated with diabetic nephropathy [70-72].

Isoforms of RAGE

RAGE exists in multiple isoforms. Full-length RAGE (fl-RAGE), a transmembrane receptor expressed on endothelial and inflammatory cells, binds circulating AGEs and activates downstream signaling cascades, including PKC, NF-κB, NADPH oxidase-mediated ROS generation, and MAPK pathways [6,73-75]. These events promote sustained inflammation, oxidative stress, endothelial dysfunction, and ECM remodeling, thereby driving the progression of diabetic retinopathy, nephropathy, and neuropathy [6,75].

In contrast, soluble RAGE (sRAGE) and endogenous secretory RAGE (esRAGE), also known as truncated RAGE, generated through proteolytic cleavage of membrane-bound RAGE or alternative splicing of the AGER gene, act as circulating decoy receptors that sequester AGEs and attenuate AGE-RAGE-mediated signaling [6,74,75]. Reduced circulating levels of sRAGE and esRAGE have been associated with heightened oxidative stress, vascular inflammation, and increased susceptibility to diabetic microvascular complications [6,75]. Furthermore, genetic polymorphisms within the AGER locus modulate transcriptional activity, ligand-binding affinity, and alternative splicing, thereby altering the balance between proinflammatory fl-RAGE and protective sRAGE isoforms and contributing to interindividual variability in disease risk and severity [6,74,75].

RAGE gene polymorphisms and associated clinical outcomes in T2DM

RAGE, encoded by the AGER gene on chromosome 6p21.3, plays a central role in mediating inflammatory and oxidative pathways implicated in the development of diabetic complications by activating NF-κB and related signaling cascades [22,62,76]. All these polymorphisms are summarized in Table 1. The rs1800624 polymorphism is located in the AGER gene promoter and influences transcriptional activity [22,62,77]. The A allele of this gene is associated with increased RAGE expression [22,77]. Clinical studies have demonstrated a significant association between the rs1800624 A allele and an increased risk of diabetic nephropathy and retinopathy [22,62,77]. The rs1800625 polymorphism is located in the AGER gene promoter and modulates transcription of inflammatory genes [78,79]. The C allele is associated with elevated circulating inflammatory markers and increased susceptibility to T2DM [78]. This polymorphism is linked to accelerated progression of diabetic nephropathy [78,79]. The rs2070600 polymorphism results in a missense mutation in exon 3 of the AGER gene, leading to a glycine-to-serine substitution at codon 82 [80,81]. This structural alteration increases the ligand-binding affinity of RAGE and reduces circulating levels of sRAGE, thereby amplifying AGE-RAGE-mediated inflammatory signaling [80]. Recent studies have identified rs2070600 as a strong genetic determinant of diabetic retinopathy, nephropathy, and cardiovascular disease in patients with T2DM [80,81]. The intronic rs184003 polymorphism in the AGER gene affects RAGE expression by regulating mRNA splicing [22,78,82]. This polymorphism is associated with increased prevalence of microvascular complications and inflammatory burden in patients with T2DM [22,82]. The rs3134940 polymorphism in the 3′ region of the *AGER* gene affects posttranscriptional regulation of RAGE expression [22,78,83]. This polymorphism leads to reduced sRAGE concentrations and endothelial dysfunction, contributing to the progression of diabetic nephropathy [78,83]. The intronic rs9469089 polymorphism modulates the alternative splicing of sRAGE and increases the risk of diabetic microvascular complications [78,81].

**Table 1:** RAGE gene polymorphisms and associated clinical outcomes in T2DM RAGE: receptor for advanced glycation end products; sRAGE: soluble receptor for advanced glycation end products; SNP: single-nucleotide polymorphism; rs ID: reference SNP cluster identification; T2DM: type 2 diabetes mellitus; NF-κB: nuclear factor kappa-light-chain-enhancer of activated B cells; mRNA: messenger RNA

SNP (rs ID)	Genomic location	Nucleotide/amino acid change	Functional effect	Associated clinical outcomes	References
rs1800624	Promoter region	-374 T → A	Reduced transcriptional activity of RAGE and lowered RAGE expression	Increased risk of diabetic retinopathy and nephropathy, and attenuated inflammatory signaling	[22,62,77]
rs1800625	Promoter region	-429 T → C	Increased promoter activity of RAGE and enhanced RAGE expression	Increased susceptibility to diabetic nephropathy and vascular inflammation	[78,79]
rs2070600	Exon 3	Gly82 → Ser (missense)	Increased ligand-binding affinity of RAGE and enhanced NF-κB activation	Increased risk of diabetic retinopathy, nephropathy, and cardiovascular disease	[80,81]
rs184003	Intron 8	G → T	Alters mRNA stability and splicing efficiency	Increased risk of microvascular complications and inflammatory burden	[22,78,82]
rs3134940	3′ untranslated region	A → G	Influences mRNA stability and posttranscriptional regulation	Reduced sRAGE concentrations and endothelial dysfunction trigger the progression of diabetic nephropathy	[22,78,83]
rs9469089	Intron region	C → T	Modulates alternative splicing of sRAGE	Reduced sRAGE levels and increased risk of diabetic microvascular complications	[78,81]

Interaction between AGEs and genetic polymorphisms in T2DM progression

Chronic hyperglycemia provides biochemical substrates for AGE formation, and the genetic polymorphisms modulate the magnitude of AGE-mediated cellular injury in patients with T2DM [9,84]. Genetic polymorphisms regulate AGE formation and detoxification pathways, thereby affecting tissue AGE burden and susceptibility to microvascular damage [9,78,85]. Variants in AKR1B1 and GLO1 genes alter intracellular concentrations of reactive dicarbonyl intermediates, thereby accelerating AGE accumulation in diabetic individuals with genetic predisposition [78,86]. Reduced glyoxalase activity due to unfavorable GLO1 polymorphisms leads to increased carbonyl stress and AGE formation in diabetic individuals [87,88].

The pathogenic impact of AGEs is further magnified by polymorphisms in the AGER gene, which regulate RAGE expression, ligand affinity, and intensity of downstream signaling [21,78,89]. High-risk RAGE variants, such as the Gly82Ser polymorphism, facilitate AGE-RAGE binding and NF-κB-mediated inflammatory signaling, thereby increasing microvascular inflammation and oxidative stress [89,90]. This genetic amplification of AGE signaling provides a mechanistic explanation for interindividual variability in microvascular complication risk [21,78,90]. Polymorphisms in oxidative stress-related genes synergize with AGE accumulation to exacerbate microvascular injury [91,92]. Antioxidant enzymes, such as catalase, SOD2, and GPX1, impair cellular redox buffering capacity, thereby intensifying AGE-induced ROS generation and oxidative damage [40,47,71]. The AGE-RAGE signaling and SNP establish a self-reinforcing cycle of redox imbalance and inflammation that accelerates microvascular complications in diabetic individuals [93,94].

Endothelial dysfunction represents a critical downstream consequence of AGE-genotype interactions [7,21,65,95]. In genetically susceptible individuals, AGEs exacerbate capillary occlusion, endothelial activation, and leukocyte adhesion, thereby accelerating ischemic tissue injury and microvascular damage [5,9,95]. Polymorphisms in the eNOS gene (NOS3) reduce NO bioavailability, leading to AGE-mediated impairment of vasodilatory and antithrombotic functions [96-98]. The interactions between AGEs and genetic polymorphisms are critical determinants of disease progression in T2DM, integrating metabolic, inflammatory, and genetic aspects into a unified pathogenic framework [21,95,97]. This AGE-SNP interaction emphasizes differences in clinical outcomes observed in patients with T2DM and reinforces the need for precision medicine strategies that integrate genetic and metabolic risk assessment to delay the development of diabetic microvascular complications [7,9,21,65].

Clinical implications and therapeutic perspectives

AGEs and genetic polymorphisms are the primary causes of diabetic microvascular complications. This interaction warrants a paradigm shift from a glycemia-centric approach to a more precision-based, mechanistically informed approach. Nonglycemic factors such as AGE load, oxidative stress, chronic inflammation, and genetically encoded vulnerability play a significant role in the development of microvascular complications among diabetics despite adequate glucose control [47,81,92,98].

AGEs have emerged as both biomarkers and mediators of T2DM progression and its microvascular complications [9,71,99]. Circulating and tissue AGE levels correlate with the severity of diabetic retinopathy, nephropathy, and neuropathy, rendering them valuable prognostic indicators for microvascular risk stratification [47,99]. Genetic polymorphisms further refine clinical risk prediction by evaluating interindividual variability in susceptibility to complications [9,47,99]. Variants in genes that regulate AGE formation, detoxification, receptor signaling, oxidative stress, and endothelial function modulate disease trajectory independently of glycemic exposure, thereby providing a molecular basis for personalized risk assessment [9,71,98]. Incorporating genetic profiling into clinical practice can identify patients predisposed to aggressive microvascular disease and tailor preventive strategies accordingly [47,98,99].

Therapeutically, targeting AGE formation and accumulation represents a rational adjunct to conventional glucose-lowering therapies. Pharmacological agents that inhibit AGE formation, such as carbonyl scavengers and dicarbonyl stress modulators, have demonstrated efficacy in reducing AGE burden and ameliorating vascular dysfunction in experimental models [71,99,100]. Interruption of AGE-RAGE signaling constitutes another promising therapeutic avenue [99-101]. RAGE antagonists, sRAGE analogs, and downstream signaling inhibitors have shown the capacity to attenuate inflammation, oxidative stress, and endothelial dysfunction in preclinical studies [62,101,102]. Modulation of this pathway may be beneficial in genetically susceptible diabetic individuals harboring high-risk RAGE polymorphisms, thereby aligning therapeutic intervention with genetic risk architecture [9,47,71].

Future research directions

Despite our understanding of the function of AGEs and genetic variants in the pathophysiology of diabetic microvascular complications, there remain knowledge gaps that require focused research. These future researchers must employ integrative, multidisciplinary methods to understand the complex interactions among metabolic, genetic, epigenetic, and environmental factors that shape the course of T2DM. A comprehensive study of the genomic architecture underlying AGE-related pathways is an important avenue. Although multiple susceptibility loci have been identified through candidate gene and GWAS studies, their individual effect sizes remain small and insufficient to fully explain clinical variation. Extensive multiethnic genomic investigations, in conjunction with thorough phenotyping, are necessary to identify rare variants, gene-gene interactions, and population-specific polymorphisms that influence AGE production, detoxification, and RAGE signaling. These initiatives will improve the capacity of genetic discoveries to be translated into clinically useful risk assessment instruments. Future studies must focus on lifestyle-genotype interactions, such as dAGE exposure, physical exercise, and environmental stressors, to understand how modifiable factors interact with genetic vulnerability to influence long-term microvascular outcomes. Clarifying these relationships could help develop tailored preventative measures to support pharmacological treatments.

## Conclusions

AGEs and genetic polymorphisms constitute an interrelated pathogenic pathway in the onset and development of microvascular complications in T2DM. Excessive AGE buildup is facilitated by prolonged hyperglycemia and acts as an active molecular driver of vascular injury through endothelial dysfunction, amplification of oxidative stress, protein cross-linking, and inflammatory activation. Genetic polymorphisms modify AGE production, detoxification, RAGE-mediated signaling, and downstream cellular responses. The degree and duration of AGE-induced tissue damage are significantly influenced by genetic polymorphisms that regulate endothelial homeostasis, oxidative stress, and AGE-related pathways. The increased interindividual heterogeneity observed in diabetic retinopathy, nephropathy, and neuropathy, despite similar glycemic control, can be explained by the synergistic interaction between elevated AGE burden and high-risk genetic variants, which amplifies inflammatory and redox signaling, reinforces metabolic memory, and accelerates irreversible microvascular damage. The convergence of genetic polymorphisms with AGE formation provides a cohesive framework for understanding the complexity of diabetic microvascular complications. It provides a solid justification for creating precision-based treatment strategies for T2DM and its microvascular complications.
